# A ‘good death’ needs good cooperation with health care professionals – a qualitative focus group study with seniors, physicians and nurses in Germany

**DOI:** 10.1186/s12904-024-01625-x

**Published:** 2024-12-20

**Authors:** Laura Mohacsi, Lena Stange, Saskia Höfig, Lisa Nebel, Daniel Broschmann, Eva Hummers, Evelyn Kleinert

**Affiliations:** 1 Department of General Practice, University Medical Center Göttingen, Humboldtallee 38, Göttingen, 37073 Germany; 2 Faculty VI - Medicine and Health Sciences, Department of Health Services Research, Division of Ethics in Medicine, Carl von Ossietzky University of Oldenburg, Ammerländer Heerstr. 114-118, 26129 Oldenburg, Germany; 3 Department of Psychosomatic Medicine and Psychotherapy, University Medical Center Göttingen, Von-Siebold-Str. 5, Göttingen, 37075 Germany

**Keywords:** Acceptance of death, Avoidance of death, Communication, Conflict, Cooperation, End-of-life care, Focus groups, Good death, Medical futility, Palliative care

## Abstract

**Background:**

Studies investigating notions of a ‘good death’ tend to focus on specific medical conditions and specific groups of people. Therefore, their results are often poorly comparable, making it difficult to anticipate potential points of conflict in practice. Consequently, the study explores how to achieve a good death from the perspective and experience of physicians, nursing staff, and seniors. The aim of this study is to identify comparable notions of a good death among the participants and to determine factors that may promote or prevent a good death, including those that may lead to futile care.

**Methods:**

The study used a qualitative design with a total of 16 focus group discussions, 5 each with physicians and nursing staff, and 6 with seniors at least 75 years old. The group size ranged between 3 and 9 participants. Analysis was carried out using Qualitative Content Analysis.

**Results:**

Three major aspects affect the quality of death: (1) good communication and successful cooperation, (2) avoidance of death, and (3) acceptance of death. While successful communication and acceptance of death reinforce each other, successful communication counters avoidance of death and vice versa. Acceptance and avoidance of death are in constant tension. Additionally, the role of family and loved ones has been shown to be crucial in the organization of dying (e.g. communicating the patient’s wishes to health care professionals).

**Conclusions:**

Communication and cooperation between patients and all involved caretakers determines quality of death. However, communication depends on several individual and organizational factors such as the personal level of acceptance or avoidance of death and the availability of institutionalized communication channels crossing professional and organizational boundaries. Furthermore, treatment cultures and organizational structures in hospitals and nursing homes often default towards life prolongation. This carries significant potential for problems, particularly because physicians emphasized the need to prevent hospital admissions when no further life-sustaining treatment is desired. In contrast, nurses and seniors were less aware that hospitals may not be the most suitable place for end-of-life care. This, along with the ambivalent role of nursing homes as places of death, holds potential for conflict.

**Trial registration:**

German Clinical Trials Register: DRKS00027076, 05/11/2021.

**Supplementary Information:**

The online version contains supplementary material available at 10.1186/s12904-024-01625-x.

## Background

Although end-of-life (EOL) wishes vary individually and can remain dynamic over the course of an illness [1, 2], both quantitative and qualitative studies have identified some universal criteria for a ‘good death.’ The most frequently mentioned criteria include pain and symptom control, clear decision-making, preparation for death, a sense of closure or life completion, being recognized as a person, and having something to give to others [1, 3–5]. Less frequently mentioned are religiousness/spirituality, a sudden death and a death at home [2, 3]. Conversely, criteria for a ‘bad death’ include inadequate or absent pain and symptom control, dying too quickly or too slowly, a lack of dignity, or disrespect toward the dying person [6, 7], as well as a lack of opportunities to plan in advance, resolve conflicts or family issues, or say goodbye [4]. Some of these criteria, such as the desire for a sudden death and the request for advance planning opportunities, appear to be inconsistent. However, it remains unclear whether these inconsistencies arise from methodological and terminological differences, or if they reflect the inconsistent nature of desires for death, which are often accompanied by simultaneous desires to live [8].

Criteria for a good death vary with gender, age, social integration, and cultural background [1, 9–11]. There is data suggesting that women oppose overtreatment stronger than men and are more likely to engage in EOL planning [10]. With age, not becoming a burden to relatives or society seems to continually gain importance [10], and trust in authority seems to be higher among older patients (median age 77), leading them to delegate decision-making to family members [12]. Depending on the country of origin, migrants may show a higher reluctance to talk about death to their families and cultural differences can cause difficulties in the communication with medical staff. One’s cultural background can also affect the orientation towards collectivistic or individualistic norms, which are associated with different decision-making models [9]. A recently published study identified loneliness as a risk factor for symptoms like anxiety, breathlessness, or pain at the EOL, while a larger social network increased the likelihood of receiving hospice or palliative care [11].

Evidence suggests that notions of a good death differ across groups: While patients and families often value psychological and spiritual aspects of EOL care as much as physiological aspects, physicians tend to prioritize physiological or biomedical concerns [4] (e.g. symptom control or biomarkers). The nursing staff’s focus on patient comfort may at times conflict with physicians’ treatment decisions [2], and there is also a divergence in the temporal orientations of staff: while physicians generally tend to focus on the patient’s future, nurses tend to concentrate on the patient’s physical and mental wellbeing in the present [13]. Lastly, patients and their families may differ in the importance they place on different aspects. In a quantitative study, family members more frequently identified life completion, quality of life (QOL), dignity, and the presence of family as significant factors. In contrast, patients were more likely to emphasize religiousness/spirituality as an important factor [3, 14].

Beyond these differences, the question arises to what extent the criteria for a good death are met in practice. Two aspects suggest that meeting these criteria is often challenging in medical practice. First, physicians and nurses have reported a lack of knowledge about EOL care and to feel inadequately prepared by their medical education to handle the emotional and professional challenges associated with patient death [15–18]. Second, numbers suggest a relatively high amount of potentially unwanted EOL hospitalizations: although only 6% of people state a preference for dying in hospital, nearly 50% die in hospital, and only 20% of people die at home, despite a majority of 76% expressing a preference to do so [19–21]. Having a high potential to become a burden for patients and their families, EOL hospitalization in some cases is used as an indicator of lower quality of EOL care [22]. Accordingly, dying at home appears as an indicator for a good death [23]. Very old age, living in a nursing home, having dementia or an oncological disease lower the probability of being hospitalized at the EOL, while multimorbidity, living in a less privileged neighborhood, or many previous hospitalizations increase the likelihood of EOL hospitalizations. Moreover, discussions about treatment goals and imminent death increase the likelihood of meeting the patient’s wishes and the criteria for a good death [24, 25]. However, these discussions are often initiated too late [25] and different medical disciplines have developed different traditions for discussion of treatment goals at the EOL that are unevenly beneficial to the patients [2, 26]. In palliative care units, there is evidence that discussions are typically initiated by physicians [13]. This may be one reason why integration in palliative care generally increases the likelihood of meeting the criteria for a good death, while intensive care unit (ICU) admissions near EOL is associated with non-compliance with the patient’s wishes or needs [25].

The number of patients receiving palliative care has increased in both inpatient and outpatient care settings in Germany [19, 21]. However, the estimated need for palliative care is not nearly covered [21] and since the development of palliative care was closely linked to oncology, the majority of palliative care patients have received an oncological diagnosis. In other medical fields, such as neurology or cardiology, the provision of palliative care remains insufficient [2, 21, 27]. There are data suggesting that whether or not a patient receives palliative care—in addition to the diagnosis and medical speciality—depends on personal dispositions and relationships of the treating physicians [15, 28]. For clinicians, the transition from curative to palliative care is the greatest barrier to EOL care [15]. This might be due to low accuracy of prognosis of remaining life expectancy [29] and physicians’ reservations and a lack of knowledge about palliative care: in acute care hospitals, palliative care is often equated with withdrawal or rejection of life-sustaining treatment (LST), without recognizing the role of palliative care for symptom management or addressing the patient’s psychosocial needs [15, 30]. Witham and Hockley suggest that this barrier to EOL care may be particularly high for cardiovascular and respiratory diseases, which are difficult to predict and therefore less likely to be diagnosed as terminal. This puts patients at risk of receiving futile care, especially the very old and frail [24].

In summary, there are individual and role-specific differences in the notions of a good death. Despite these differences, universal criteria for a good death and conditions that promote or hinder these criteria are apparent. However, it remains unclear how role-based differences affect the fulfillment of these criteria. This may be linked to weaknesses in assessing whether criteria are met: dying in an undesired place can impact the quality of death, but hospitalization does not inherently equate to bad dying, nor does dying at home guarantee a good death. In addition, many factors that may determine the quality of death, such as hospitalization or involvement in palliative care, depend on the respective national health care system. While numerous studies have been conducted on this topic, data available for Germany is limited. Furthermore, most studies include only one or two participant groups, with specific inclusion criteria like a certain diagnosis or affiliation with a medical discipline or hospital ward. Given the variability in perceptions of a good death, this focus seems justified. Nevertheless, the comparability of results is low, and important interrelations, like cross-organizational cooperation, are at risk to be neglected. The perspectives of seniors without severe medical diagnoses, but who are preoccupied with death due to their age, are marginalized. As noted, seniors are particularly affected by issues of EOL care.

Therefore, this study explores perspectives of senior citizens and of physicians and nursing staff regularly treating people of old age on how a good death can be achieved. The aim of the study is to obtain results that will allow the three groups to be compared and to identify factors that may promote a good death as well as the potential for conflict or dissatisfaction that may prevent a good death.

## Methods

### Design

The study employed a qualitative research design. Focus group discussions were conducted with people of old or very old age (at least 75 years), as well as with medical and nursing staff who regularly treat people of old age. The data collection was carried out within the framework of the research project “Medicine in older age—perception and assessment of ageing processes by older people and medical and nursing professionals.” The project is part of the DFG research group “Medicine, Time and the Good Life.” A study protocol, including detailed information on the study’s procedure, has already been published [31]. For the purpose of this paper, all passages relating to death have been analyzed. The analysis was conducted using Qualitative Content Analysis according to Kuckartz [32]. Following the methodological tradition of qualitative interpretive social research [33], it was assumed that individual notions of a good death are intertwined with social norms. For the presentation of methods and results, the researchers adhered to the Consolidated Criteria for Reporting Qualitative Studies (COREQ) [34]. Participant’s quotes were translated from German into English by LM. False starts and non-lexical expressions were removed in the translations to improve readability and save space.

### Participants

**Fig. 1:**
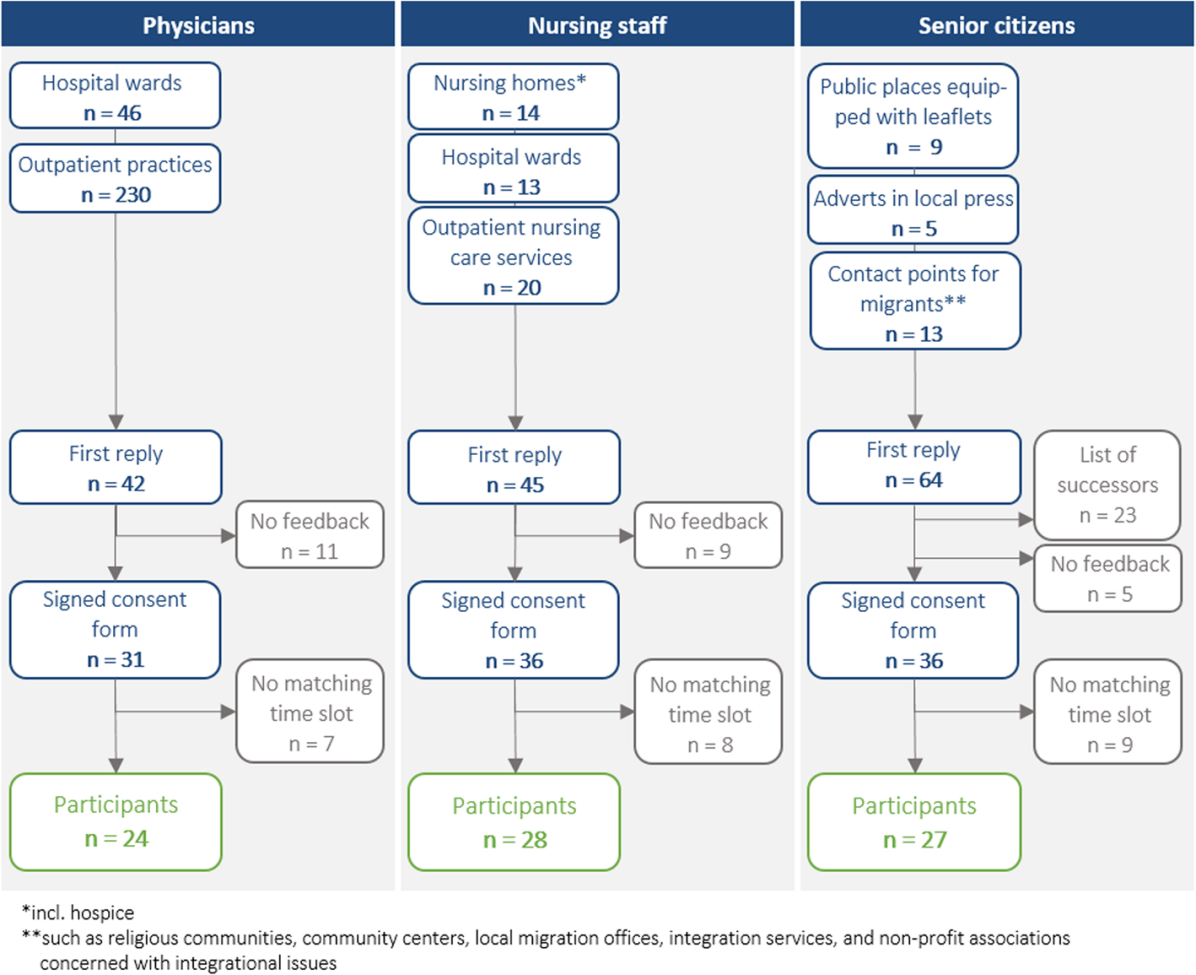
Recruitment process of participants (*N* = 79)

To recruit senior citizens, the researchers distributed a press release in local newspapers, along with leaflets and posters in senior centers, community centers, and educational, sports, and cultural institutions (see Fig. 1). Efforts to include seniors with a migration background by contacting migrant centers were unsuccessful. Study information, response forms, and consent forms were sent to interested individuals. Participants were selected based on age, gender, education, and subjective health status to ensure diverse group compositions. Self-reported health status was assessed using two global questions from the SF-12 (a short version of the SF-36 health survey) [35]. A total of 27 seniors participated in six focus group discussions.

For medical and nursing professionals, convenience sampling was used. However, additional purposive sampling was used to ensure representation of different institutions, disciplines, and professionals from outpatient and inpatient setting. The researchers utilized the department’s network to contact nursing homes, outpatient nursing care services, hospital wards, and outpatient practices, requesting distribution of study materials among their employees. As part of a subsequent snowball sampling, all contacted persons were encouraged to further disseminate the material. In total, 28 nurses and 24 physicians participated, with drop-outs due to availability, time constraints, or lack of response (Fig. 1). Table 1 provides demographic details of all participants. The composition of the focus groups can be found in additional file 1.

**Table 1:** Self-reported socio-demographic and professional characteristics of participants (*N* = 79)

		Physicians (*n* = 24)		Nursing staff (*n* = 28)	Seniors (*n* = 27)
Gender	female male predominantly male	14 10 -		18 10 -	17 9 1
Age (y)	mean (SD) range n.a.	47.0 (10.5) 27–67 2		45.8 (13.5) 25–69 -	79.8 (4.0) 75–89 -
Sector^a^	inpatient outpatient	13 12		20 8	
Professional experience (y)	mean (SD) range n.a.	19.2 (10.8) 1–43 2		17 (10.2) 1–42 -	
Discipline^b^	general practice internal medicine neurology orthopedics / rehabilitation other supplementary training in palliative care supplementary training in geriatrics		7 6 3 4 4 3 3		
Living situation	single household living with partner family home / shared flat assisted living care facility				14 10 2 1 -
Health status (self-reported)	excellent very good good less good poor				1 5 16 5 -

^a^Participants involved in both inpatient and outpatient care were accounted to both categories

^b^When participants specialized in more than one discipline, main occupation at the time of data collection was counted

Appointments for group discussions were arranged via phone, email, or mail, with researchers emphasizing openness to queries.

### Data collection

Data were collected between May 2022 and August 2023 through 16 focus groups, six with senior citizens and five each with medical and nursing professionals. The focus groups with physicians, nurses, and seniors were conducted separately to enhance comparability between participant groups, and to prevent power imbalances, ensuring that all participants could speak openly. All focus groups lasted one to two hours. One nursing group and three physician groups were conducted online via BigBlueButton, a video-call platform consistent with the data protection requirements of the department, while the rest were held in the department’s conference room. Moderation alternated between LM, EK, and SH, with at least two of them, but no others, present at each session.

The focus group guide (additional file 2), developed by EK, LM, and LS based on the method of Helfferich [36], was piloted in the first discussion with each participant group, after which EK and LM made adjustments if necessary. As case vignettes provide an impulse to generate narratives that reflect larger societal patterns of interpretation [37], EK and LM developed two case vignettes in collaboration with medical colleagues, which were later reviewed with EH. One vignette specifically provoked discussions about the refusal of LST (additional file 3), ensuring that the topic of death was addressed in every group discussion. Participants were encouraged to contribute own experiences. Immediately after each focus group, EK and LM discussed main themes and differences from previous groups, ensuring data saturation was acknowledged when indicated by a lack of new main themes.

Participants provided written consent for audio recording, which was transcribed verbatim using f4x software and manually corrected by the research team (LM, SH, EK). Researchers also took field notes to capture observations and the ambiance, though these were not systematically analyzed. Transcripts were not returned to participants.

### Analysis

Data were analyzed using Qualitative Content Analysis following Kuckartz, a summarizing method that captures both the manifest and latent meanings of the data [32]. EK, LM, and SH developed a code tree (additional file 4) for each participant group using MAXQDA software, generating codes mainly inductively. Data coding was conducted independently by EK, LM and SH, followed by reconciliation through consensual coding [32], with LS participating in regular meetings. EK, LM and SH systematically compared the code trees across the three participant groups. The findings were presented to the research group “Medicine, Time and the Good Life” at different points in time. Participants did not provide feedback to the findings, they will receive a summary.

## Results

**Fig. 2:**
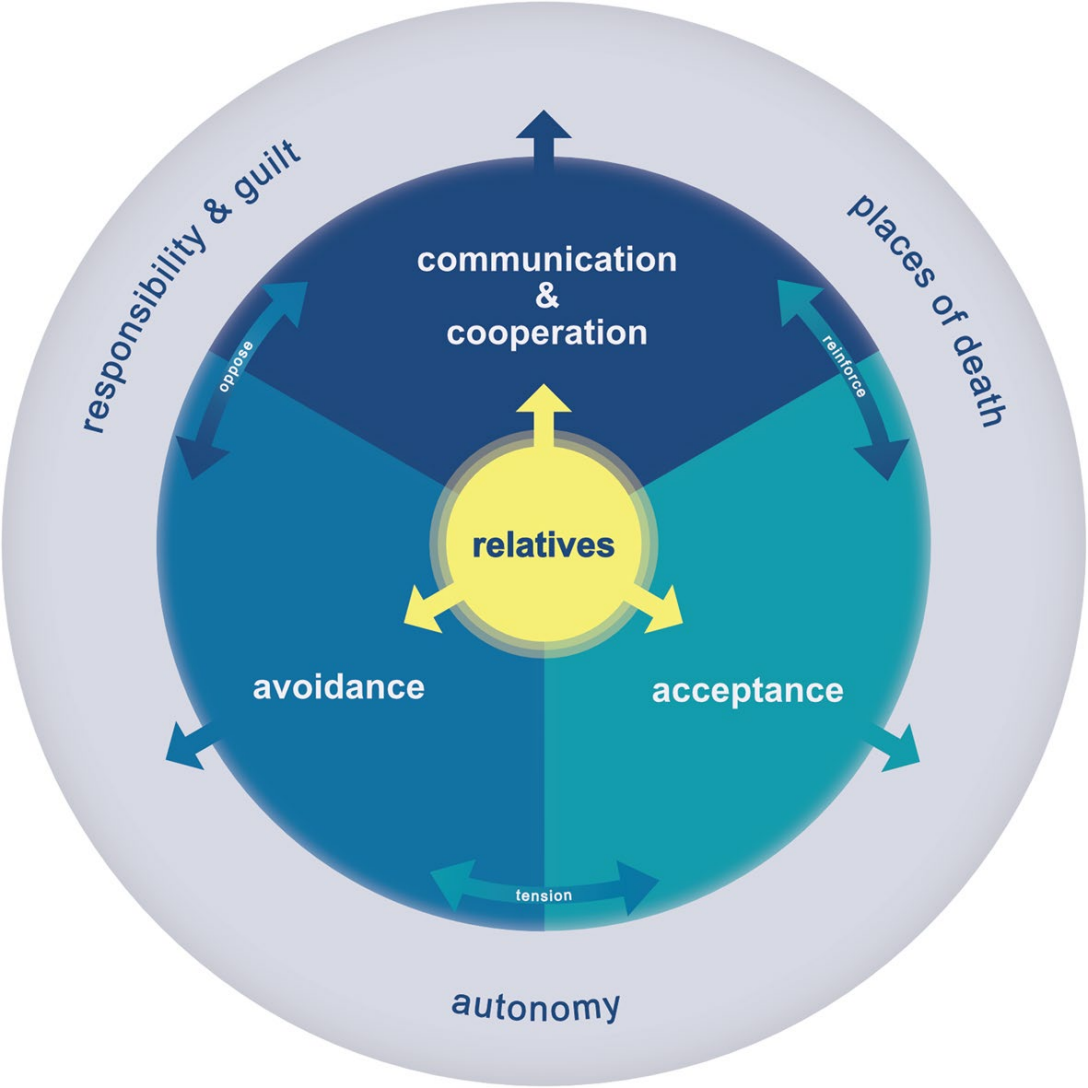
Main categories (blue circle), minor themes (grey circle) and their relations (read from inside to outside of the circle)

Three main factors impacting the quality of death have been derived from the data: (1) successful communication and collaboration, (2) acceptance of death, and (3) avoidance of death. While successful communication and acceptance reinforce each other, communication stands in opposition to avoidance of death. Acceptance and avoidance of death are in constant tension (Fig. 2).

The role of relatives was crucial across all categories. Relatives are integral to a good death, involved in EOL decisions as caregivers, advocates, and as those who have to bear the consequences of medical interventions. Participants of all groups identified not only as representatives of their respective group, but also as relatives, adopting the perspective of relatives. This highlights that institutional role players remain integrated into social relations shaping their experiences and attitudes. Professionals shared personal experiences alongside their professional ones, and some seniors even judged the quality of death based on how well relatives could cope. Therefore, relatives do not form a separate category but are intertwined with the three main categories (Fig. 2).

Three recurring patterns of argumentation were conceptualized as minor themes: (a) places of death, (b) responsibility and guilt, and (c) the ideal of autonomy (Fig. 2).

### Successful communication and collaboration

Communication around death is multifaceted. Participants described various aspects, including preparation (ranging from years in advance to just before death), eliciting and establishing the patient’s wishes, mediating between parties, enforcing those wishes, agreeing on preferences, and deciding on treatment. Patients, their families, physicians, nurses, and other health care professionls (HCPs) involved were identified as key stakeholders in this communication.

#### Seniors

As a value, clear communication was mentioned only occasionally by the seniors. However, all of the stories in which the senior participants portrayed death as ‘good’ were also stories of successful communication and collaboration with the HCPs involved.

I was very grateful that our GP said we had to respect her wishes. Yes, he had already told me weeks before that if anything happened to her, ‘don’t call an ambulance. Here you have my mobile number.’ Yes. ‘We all know that your sister doesn’t want to go to hospital.’ […] Yes, and of course I felt really well supported.

*Female*,* 80*,* seniors group 2*,* line 545–551*.

Conversely, all stories in which death was portrayed as agonizing, painful, and distressing were stories of failed or missing communication or unsuccessful collaboration between patient, families, and HCPs.

#### Physicians

Physicians held an ideal of consensus. This involved reconciling patients’ wishes with those of their families, medical indications, feasibility, and ethical acceptability. Consensus was seen as something to be achieved not only with patients and their families, but also with colleagues and other HCPs. Some physicians found it helpful to consult their team when facing a difficult decision. The idea of consensus was closely associated with the physicians’ duty to provide patients with adequate information about their health status and medical options, including possible risks and benefits, and to explore alternatives together with patients and their families. The physicians’ awareness of their own responsibility was reflected in their demands for an honest dialogue with the patient and for shared decision-making (SDM).

It’s a typical revolving door phenomenon, you could almost call it that. The patient eats, goes to hospital, pneumonia, is treated, comes back, eats again, back to hospital, pneumonia. And from my point of view, something is going completely wrong. So I think there should have been a dialogue much earlier. What is supposed to happen? What should the future look like?

*Male*,* 38*,* neurologist*,* outpatient & inpatient care*, *physicians group 3*,* line 429–434*.

The physicians also reported structural obstacles that hinder communication and collaboration between colleagues, particularly across professions and sectors. They described a lack of institutionalized communication channels between inpatient and outpatient care, leading to treatment possibly unwanted by the patient.

#### Nursing staff

The nurses felt a strong sense of proximity to patients, spending more time with them than physicians. They reported frequent communication with patients, believing they understood their needs and wishes better than physicians. This led them to see themselves as mediators between patients and physicians, and sometimes between patients and their families. In contrast to patients and family members, nurses highlighted their ability to make forward-looking decisions due to medical knowledge. Compared to physicians, they emphasized that their lower responsibility in decision-making reduced their fear of making mistakes. This, in their view, enabled them to make what they considered more humane decisions.

We nurses have to bear this [the consequences of the decision; LM], but we are not the first authority in the decision, so to speak. We are involved because we are at the bedside and know the patients better, including their characteristics. But we have to bear it, yes. Even if we are against it. I always say that I think nurses have a bigger heart than doctors who only come to the bedside three times a day.

*Female*,* 66*,* former ICU nurse*,* retired*,* nurses group 1*,* line 108–112*.

The nurses noted a power imbalance between themselves and physicians, which they found particularly problematic given their mediating role. This power imbalance was also evident in their experiences as family members, making it difficult to assert a patient’s will against physicians’ recommendations. Especially because they often had the impression that their opinions and assessments were not listened to, they emphasized the importance of a well-functioning team for effective communication. Poor communication was associated with low job satisfaction, ultimately to the detriment of patients. They also reported family conflicts when communication about a family member’s EOL had failed or been absent.

### Avoidance of death

All groups reported various tendencies to avoid, prevent, deny, or repress death. For simplification, these tendencies will be summarized here as avoidance of death, though it only partially covers cognitive and psychological aspects. Avoidance was observed at individual, familial, societal, and organizational levels. Participants described family members, friends, or acquaintances who preferred not to think or speak about death, and criticized a general lack of interest in and knowledge about EOL issues. Within families, specific barriers to discussing death were experienced from both patients’ and relatives’ perspectives. At the organizational level, participants identified treatment cultures and structural obstacles that prevent a good death, including instances where individuals were ‘not allowed’ to die.

#### Seniors

Among the seniors, this was expressed as ‘having to fight to be allowed to die in peace.’ They recalled numerous instances of HCPs attempting to prevent the death of family members or loved ones, often despite it was known that no further treatment was wanted. From a relatives’ perspective, such futile care was distressing. To ensure a peaceful death, families had to defend and enforce the patient’s wishes against hospital or nursing home staff.

[…] [T]hat man is […] 88, has had several strokes a long time ago, has dementia, but is still relatively mobile, and his wife takes good care of him. Hospital, curative treatment, I don’t know what they wanted to do. And when I got there one day, and they wanted to wake him up and feed him, I said, ‘well, if I were to look at this from a palliative care perspective, I would let him sleep now.’ […] So they tried everything, and then we managed, together with his wife, to reach the doctor and convince her, ‘I think he’s dying.’ […] And then he was allowed into a single room and they stopped all therapies they had planned and he was allowed to die, in peace and quiet. […] I mean, all the things you have to get going sometimes. That’s disgusting, isn’t it? Just because it’s not recognized that that man is dying.

*Female*,* 75*,* voluntary EOL companion*,* seniors group 1*,* line 409–439*.

The quote highlights the effort patients and their families must make to counter institutional tendencies to prevent death. However, the seniors had noted that family members hold on to the patient’s life, regardless of the patient’s wishes. Similarly, the seniors reported difficulties in discussing their own deaths with their children, as they were reluctant to speak about death.

#### Nursing staff

Nurses emphasized EOL decisions made deliberately against the patient’s will. One of the reasons given was the inability of families to accept the imminent death of a loved one. The nurses had experienced numerous cases in which family members were unable to let a patient go. In some cases, this led to a life prolongation the patient did not want. As a second reason, the nurses named physicians’ reluctance to terminate curative treatment. This was linked to financial interests, reluctance to take ethical responsibility, or fear of liability.

Hospitals often just give me the medical view and say: ‘now, the somatic condition is like this. If nothing is to happen, the safe option would be the feeding tube.’ They give this to the family and these counseling sessions, they are always very one-sided. […] The patient’s will, their pleasure in eating, takes a back seat and if they are not an energetic person who asserts themselves and are also mentally able to stand up for their wishes, then it is not uncommon for this to be imposed or decided for them. So that no one feels guilty about having done something wrong.

*Male*,* 43*,* nurse in inpatient geriatrics*,* nurses group 3*,* line 148–157*.

Thus, the nursing staff critically described treatment cultures and organizational structures in hospitals and nursing homes that impede a good death. However, these obstacles were depicted as highly dependent on the specific institution, care unit, or single staff members, and a decline in these tendencies to prevent death had been observed in recent decades.

#### Physicians

Similar to the nurses, the physicians noted difficulties to deactivate implemented devices, to withdraw already initiated treatments or to reject available treatment options. Such difficulties are part of a treatment culture committed to the preservation of life.

And I think that’s always the case where a PEG [percutaneous endoscopic gastrostomy; LM] is used in nursing care, the patient no longer gets anything to eat. Yes, because that’s like putting a stop sign over their mouth and as if the mouth was sewed up. […] And these are major mental hurdles. And what I mean by multi-professional team approach is that there are simply too many emotional barriers, which simply hinder and prevent sufficient progress.

*Male*,* 53*,* specialist in internal medicine*,* outpatient care*, *physicians group 1*,* line 472–483*.

Further, the physicians noticed that patients or their family members struggled to let go. They recalled relatives withholding the patient’s will as well as patients demanding treatment in cases with low prospects of success. The cause was often thought to be anxiety originating from a lack of confrontation with death.

It gets complicated when the patient’s wishes contradict the intuition. I have a lot of oncology patients who are somehow in the seventh line and still say, ‘I want to be resuscitated,’ ‘I want to be in intensive care,’ ‘I want a tracheostomy,’ where you think to yourself, ‘why? You’ve already outlived your disease.’

*Female*,* 28*,* inpatient urology (specialist in training)*, *physicians group 2*,* line 559–564*.

However, the physicians had observed an increase in the number of patients who preferred to die at home. This was interpreted as a growing willingness in society to engage with issues of death.

### Acceptance of death

The data showed that acceptance of death must be established through social interactions and communication. Participants requested in various ways that a patient’s death should be accepted at a certain stage of the disease or when the patient wishes to die. Acceptance of death was typically demanded when there was no expectation that LST would maintain or enhance the patient’s QOL, or when the QOL was even threatened by possible LST. The call for acceptance of death was addressed to patients themselves, their relatives, treating physicians, nursing staff, and, where applicable, other HCPs.

#### Seniors

The seniors expressed the acceptance of their own death as a task involving an ‘inner cleansing’ in the sense of coming to terms with themselves. This included resolving social conflicts and addressing mental issues.

Yes, well, I think, is there anything left unfinished in life? That can be very private, like whether you still want to solve any conflicts or achieve any goals. So I think, for me, that’s part of it. […] I think that one of my goals is to be able to die well, for example. So, not with anger or accusations or anything like that. That’s an example, but it’s an inner goal.

*Female*,* 75*,* seniors group 2*,* line 1008–1014*.

Such mental and social preparation for death was seen as a task to be done at an earlier age, before periods of illness or need for care occur. People who had been able to embrace their own imminent death were referred to positively.

The seniors also demanded acceptance from HCPs, patients, and patients’ family members and friends. Quality of death was partly evaluated according to the relatives’ experience of the situation.

[He] died with Covid. So he couldn’t swallow anymore. So exactly this. Dramatic. Months of this drama, so difficult for his wife, too, I think. I would have let him die right at this moment, but you could tell he really wanted to live.

*Female*,* 75*,* voluntary EOL companion*,* seniors group 1*,* line 409–412*.

#### Physicians

The physicians held the perspective that patients need to prepare for their death. Preparation for death was typically understood as consideration and reflection leading to knowledge of one’s own treatment preferences. An advance directive was seen as the ‘gold standard’ of preparation for death. The rationale provided was partly to reduce the moral and emotional burden on physicians or relatives and to streamline decision-making.

Yes, and at the age of 104 she was completely overstrained by the question of whether she would want to be resuscitated, if she were lying dead in bed, so she couldn’t give me an answer. I found it fascinating how a 104-year-old couldn’t find an answer to that question yet.

*Female*,* 50*,* specialist in physical and rehabilitative medicine*,* outpatient care*, *physicians group 4*,* line 824–828*.

However, physicians were aware of their own responsibility in general and to address the issue with patients, to encourage them to identify their treatment preferences, and to co-create advance directives. Accordingly, physicians emphasized the necessity of SDM in multiple contexts.

#### Nursing staff

Similar to the physicians, the nursing staff claimed acceptance of death in the form of preparation for death. In contrast to the physicians, however, they adopted a private rather than a professional perspective, recalling instances in which they encouraged their relatives to create advance directives to avoid EOL decisions.

In terms of perceived patient proximity, the nurses addressed their claim for acceptance to patients’ families and treating physicians trying to prevent death.

A big problem is that nothing is organized in the nursing homes. […] When someone is admitted to a nursing home, there must an be arrangement made on admission, with the guardian, the children, or whoever, stating that a candle will be lit and no emergency doctor will be called.

*Male*,* 54*,* nurse in neurological ICU*,* nurses group 2*,* line 418–428*.

The quote illustrates that these claims for acceptance were usually also claims for institutionalized communication channels that facilitate acceptance of death.

### Minor themes

Minor themes highlight important patterns of argumentation that recurred across groups, though they were not as central as the main categories. These patterns often emerged in discussions surrounding the main topics and illustrate the tensions between different arguments or courses of action.

#### (a) Places of death

Different ways of dealing with death, i.e. whether death was accepted or avoided and how death was communicated, were linked to places of death. In terms of avoidance tendencies, the hospital, with exception of palliative care units, is conceived as a place of institutional prevention of death. Participants criticized organizational procedures and treatment cultures that prevented death and deprived patients of the peace and quiet necessary for a dignified death. Hospices and palliative care units were explicitly excepted from the treatment culture of preventing death. In contrast, they were conceived as places of acceptance and clear, honest communication. Physicians viewed the place of death as something that determined the medical options. In contrast to the seniors and the nurses, they implicitly assumed that the hospital was an unsuitable place to die. As they discussed, it became clear that if someone wanted to die, they would have to make sure that they were no longer going to be hospitalized.

And I’ve often experienced it, even with 95-year-olds, I had one just last week who came in with gastrointestinal bleeding and then somehow got an OGD [gastroscopy; LM] and colo [colonoscopy; LM]. Then said to me afterwards that she hadn’t really wanted to live for quite a while. And I also think that if this had been discussed properly in the nursing home, she would not have come to us in the first place […].

*Male*,* 27*,* inpatient internal medicine (specialist in training)*, *physicians group 5*,* line 699–705*.

In this sense, the hospital was seen as a place to save lives, while dying at home, where good palliative care was available, was seen as a place where patients were allowed to die. The nurses and the seniors considered it self-evident that patients and their families preferred dying at home.

As place of death, nursing homes appeared to have an ambivalent role. On the one hand, the nursing home is the patient’s home and therefore seen as a place of peaceful death. On the other hand, the nursing home is a medical institution where, at least in part, the same treatment cultures apply as in a hospital. This may lead to repeated hospitalizations and a prevention of death.

#### (b) Responsibility and guilt

The tension between accepting and avoiding death led to a constant negotiation of responsibility and guilt. The demand for advance directives reflects a desire for institutionalized criteria to guide decisions, indicating that EOL decision-making is perceived as distressing. Participants described own and third party tendencies to avoid these decisions, with all groups noting that family members often struggled to decide due to emotional or ethical concerns. Given their existential dimension, making such decisions for others was seen as burdensome. Participants reported feelings of guilt after refusing or withdrawing LST for a relative, even when it was known that this was compliant with the patient’s wishes.

When I got to work in the morning, I called the hospital […] and then one morning they told me, ‘we’ve just resuscitated your father.’ 83 years old. And so I told them, ‘please, next time don’t. I know very well he does not want it.’ I got there at midday and he had died. They had taken my telephone order. Surprisingly. I still had to go to psychotherapy afterwards because I felt responsible.

*Female*,* 76*,* seniors group 1*,* line 646–652*.

Nursing staff and physicians discussed what responsibilities family members should take on and what decisions they should be able to make. With little consensus, some felt that it was primarily the role of family members to determine the patient’s wishes, while others were uncomprehending and surprised by family members who wanted to leave the decision to the physicians.

Physicians felt responsible for both bearing the consequences of discontinuing treatment and considering the outcomes of continuing it. For example, they emphasized that oral feeding was still possible with a gastric tube, but expressed concern about whether oral feeding would actually continue, especially in a nursing home setting. For relatives, not seeking available treatment was often associated with feelings of guilt.

#### (c) Ideal of autonomy

Like responsibility and guilt, autonomy was negotiated within the tension between acceptance and avoidance of death. Communication and acceptance of death help maintain autonomy at the end of life. The belief that only the patients themselves can make EOL decisions is rooted in the concept of autonomy.

That was the case with my father as well, he had to have a heart valve operation when he was 80. He decided against it. He lived for another ten years. That’s a decision. Only the person themselves can make that decision. I can’t make that decision for him.

*Male*,* 63*,* nurse in the ICU*,* nurses group 1*,* line 230–232*.

Dying under someone else’s control, without one’s wishes being respected, was considered a bad death. However, autonomy requires certain prerequisites, above all awareness of one’s own (imminent) death as a premise to make decisions and give consent. Awareness of one’s death was part of the seniors’ ideal of ‘inner cleansing,’ and nursing staff distinguished a ‘dirty death’ from a ‘good death’ based on this awareness.

Autonomy was also called for in the context of assisted suicide. Assisted suicide and its relationship to autonomy were discussed controversially. Some of the seniors expressed a desire for assisted suicide as a legitimate means to prevent a loss of autonomy at the EOL. Physicians, however, approached the subject with caution and often responded by recommending palliative care instead.

## Discussion

The findings of this study demonstrate that effective communication among all stakeholders and collaborative decision-making are central to achieving high-quality EOL care. This was particularly evident in the seniors’ narratives which strongly associated successful collaboration with a good death. This theme was further mirrored in the physicians’ commitment to reaching consensus among all involved parties as well as in the nurses’ indignation over unmet patient wishes at the EOL. Both physicians and nurses observed treatment cultures and organizational structures that often defaulted to life-prolonging measures, potentially leading to overtreatment at the EOL. This observation aligns with the recurring narrative among seniors of “having to fight for the right to die in peace.” Overall, family members were highlighted as playing an essential role across all dimensions of a good death, often serving as advocates for patients in this process. However, family members could also hinder a good death, particularly when communication was lacking or when they struggled to let go. Role-specific differences in perceptions of a good death identified in this study align with existing research [1–3, 9, 11, 13, 14, 38, 39]. For instance, patients and their families often weigh medical, spiritual, and psychological considerations equally, whereas physicians tend to focus more on clinical aspects [4]. Additionally, the literature has documented nurses’ experiences of moral distress when required to implement treatments they view as futile [40]. The results of this suggest that power imbalances between nurses and physicians potentially exacerbate nurses’ distress and may serve as an additional burden in EOL care.

### Communication

The data of this study indicate that a good death depends on successful communication and cooperation among all parties involved in EOL care and decision-making. This aligns with findings from studies showing that discussions about EOL treatment goals increase the likelihood of a good death [24, 25]. The nurses’ sense of being in a mediating role complies with findings that nurses provide a large part of the collaboration efforts [41]. Participants in this study balanced life extension against QOL in their discussions about a good death, a consideration also noted by Rodriguez and colleagues [38].

However, the data show that good EOL communication depends on various individual and organizational factors: (1) patients’ and their families’ individual level of acceptance or avoidance of death; (2) problematic treatment cultures and organizational structures in hospitals and nursing homes; (3) a lack of institutionalized communication channels; and (4) differences in the perceptions of hospitals as a place of death across groups leading to EOL hospitalizations that may be considered futile.

### Individual level of acceptance or avoidance of death

The results of this study suggest that acceptance of death is related to the discussion of EOL issues. Data from this and other studies suggest that discussing EOL issues is often challenging for patients, their families, and HCPs [2, 25, 26]. Some studies have indicated that religious belief can help discussing and accepting death [2, 16]. In this study, religiousness or spirituality were rarely discussed by participants and played a subordinate role. However, participants were not asked about religious or spiritual beliefs and there could be a bias in the sample. Among the seniors, the primary motivation to participate was the need to discuss old age, medical care, and death. It could be assumed that this need is more prevalent among nonreligious individuals, as religious or spiritual communities often fulfill such needs.

### Problematic treatment cultures and organizational structures in hospitals and nursing homes

Beyond the challenges of discussing EOL issues, institutional factors often contribute to the avoidance of death. The problems reported in acute hospitals and nursing homes align with the frequently cited lack of knowledge and training about EOL issues and terminal care among HCPs [15–18]. Participants in this study described treatment cultures and organizational structures that, by default, prioritize sustaining life unless the patient or their advocate intervenes. Furthermore, it was shown that withdrawing LST, once initiated, can be difficult for physicians. This corresponds with findings on medical futility [42, 43] and the concept of the ‘technological imperative’ as described by Kaufman and colleagues. Using the example of implantable cardioverter defibrillators on people aged 80 or older, they describe how availability of technologies and the values ascribed to them determinate therapy. Especially physicians feel morally obliged to perform treatment when available, because it is difficult to refuse care procedures once they have become standard [44, 45]. Similarly, Hermann describes an “arrangement of hope,” with all stakeholders adhering to curative treatment goals in order to be able to maintain communication as well as physician’s agency [46]. Treatment cultures with regard to dealing with death vary with medical disciplines and hospital units [2, 26]. This is underlined by the fact that medical futility is a particular problem in the ICU [47] and completes the picture drawn by the treatment cultures found in this study. Evidence that the ‘medicalization of death,’ e.g. multiple hospitalizations, ICU admissions, or chemotherapy near death, is associated with poor quality of death [25] is also consistent with the results of this study.

Participants from all groups reported that the drastic shortage of nursing staff in Germany significantly impacts EOL care. This shortage is a well-known issue in Germany, with negative effects on the quality of care, job satisfaction, and the mental and physical health of nurses, as well as their participation in social life [48, 49]. It can be assumed that this shortage contributes to treatment cultures and organizational structures which may prevent a good death. However, since staff shortage was not a focus of this study, no further conclusions can be drawn.

### Lack of institutionalized communication channels

The data of this study suggest a lack of institutionalized communication channels crossing professional and organizational boundaries. This may be one reason contributing to the problematic treatment cultures and organizational structures named above. Consistent with this, studies indicate that interprofessional collaboration in EOL care settings in Germany needs improvement. In home care, the absence of such collaboration often forces patients and families to manage organizational tasks like forwarding information and coordinating treatment and care [41]. In nursing homes, the high rate of hospital admissions within the last year of life (about 50%) can be attributed to low levels of interprofessional collaboration, with many of these admissions being more harmful than beneficial to patients [50, 51]. However, these issues do not apply to the hospice and palliative care sectors, where communication and collaboration are known to be effective [15] and palliative care interventions combining both hospital and home care promise positive outcomes [52].

### Hospital as a place of death

While several studies have noted differences in perceptions of a good death [1–3, 9, 13, 14], this study uniquely identifies differences in perceptions of the hospital as a place of death. While seniors and nurses criticized the handling of death in hospitals, physicians naturally considered hospitals to be an unsuitable place for death and argued that those who want to die have to make sure that they are no longer admitted to hospital. To the authors’ knowledge, these differences have not been previously discussed. They highlight an important point of conflict in EOL care planning, as they reveal differing premises among stakeholders. This may help explain the discrepancy between the high rate of hospital deaths and the relatively low number of people who express a preference for dying in a hospital.

### Responsibility and guilt, autonomy

The power imbalance noted by the nurses, the seniors’ impression that they had to fight for a good death, and the shift of physicians into a relatives’ perspective are consistent with research suggesting that EOL decision-making is shaped by physicians’ value judgements [15]. However, physicians’ awareness of their own responsibility suggests that they have a feeling both for their own decision-making power and for the vulnerable position of the patients at the EOL and speaks in favor of concepts of relational autonomy as elaborated by some medical-ethical approaches [53, 54].

Feelings of guilt that arose among participants after making the decision to withdraw or refuse LST, and clinicians’ requests that patients reflect on their EOL wishes and treatment preferences, suggest that EOL decision-making can be stressful, leading to a tendency to avoid such decisions. This goes hand in hand with the idea of patient autonomy as a relieving function, i.e. responsibility is shifted to patients to relieve HCPs and family members of burdensome decisions [55]. Some approaches to medical ethics address the relief of relatives as a motivation for advance care directives and for a good death in general [56]. The senior’s demands for autonomy in form of euthanasia mirrors Streeck’s hypothesis of euthanasia as a preventive act to avoid dependency in old age [57].

### Strengths and limitations

This study provides rare and valuable data for Germany, with high comparability across the three participant groups due to the use of case vignettes. By including seniors solely on the basis of age, it comprises a group that has yet rarely been studied. The data highlight key conflicts of EOL care, including both individual and systemic aspects. Notably, all participants also reported from the perspective of relatives, effectively adding a fourth group to the study. While the use of relatives as proxies has been shown to be adequate for assessing the quality of death [2, 58], in this study, it is not possible to clearly distinguish the relatives as a separate group and compare them to the other groups.

There is likely a bias in the participant sample, as the study excluded seniors in need of long-term care or nursing home residents. Therefore, it cannot provide insights into how the perspectives of older people change as their health deteriorates and they become more dependent. The seniors included in the study had an above average socio-economic status and were homogeneous in terms of nationality, first language, and ethnicity. As a consequence, class-related aspects of EOL care and cultural differences remain unaddressed.

In contrast, the sample of professionals was more diverse. Although nationality and cultural background were not systematically documented, one physician openly discussed their migration background, highlighting cultural differences in EOL care. The nursing staff sample included a variety of cultural perspectives and perspectives from individuals with a less privileged socioeconomic status, such as nursing assistants. However, it is likely that only professionals already interested in the topic participated, and it is possible that those less interested might have different attitudes, possibly showing less responsibility or commitment to SDM.

In recent years, assisted suicide has become a frequently discussed topic in Germany, and the debate has gathered pace due to several legal changes [59]. Some of the participants addressed this topic. Given the unresolved legal situation and the virulence of the ongoing public debate on assisted suicide, this topic requires a separate methodological approach in order to be adequately investigated. Therefore, it was not systematically included in this study, although it may be an important aspect of a good death.

## Conclusions

The study was able to show three major aspects that affect the quality of death: (1) good communication and successful cooperation of stakeholders, (2) acceptance of death, and (3) avoidance of death. Analyzing their interrelations, the data revealed potential obstacles for a good death, including both individual factors and social norms embedded in the medical system through organizational procedures: (a) differing perceptions of the hospital as a place of death among stakeholders, (b) the lack of institutionalized communication channels, (c) treatment cultures and organizational structures inherently designed to prolong life, and (d) challenges within families to discuss EOL wishes and the topic of death.

Given the scarcity of data on cross-organizational obstacles and obstructive treatment cultures in Germany, these findings help fill important research gaps. The study provides critical insights into why a good death is sometimes hindered within the medical field. It underlines the importance of cross-organizational communication and cooperation in ensuring a good death. As such factors can only be achieved by comparative approaches, future research should aim for comparable results. Further, future studies should include a focus on treatment cultures and organizational structures to better understand and address these barriers.

The results of this study have important implications for clinical practice and the public respectively patients and their families. For clinical practice, the study highlights the importance of striving for effective communication to initiate involvement in palliative care at the right time. Addressing what it means to die in a hospital and how this can be avoided was shown to be of particular relevance. For patients and their families, the study highlights the value of ensuring that family members have detailed and timely information about EOL wishes and preferences. While discussing EOL wishes and the prospect of impending death with loved ones is challenging, both patients and family members are likely to benefit from these conversations.

## Supplementary Information

Supplementary Material 1.

Supplementary Material 2.

Supplementary Material 3.

Supplementary Material 4.

## Data Availability

An overview over the composition of focus groups, the focus group guide, the case vignette, and the code trees are available as additional files. Due to data protection requirements, the original data are not publicly available. However, upon reasonable request, pseudonymized transcripts may be provided by the authors.

## Abbreviations

ACP: Advance care planning
COREQ: Consolidated Criteria for Reporting Qualitative Studies
EOL: End of life
HCPs: Health care professionals
ICU: Intensive care unit
LST: Life-sustaining treatment
SDM: Shared decision-making
QOL: Quality of life
