# Supplementary material for: A ‘good death’ needs good cooperation with health care professionals – a qualitative focus group study with seniors, physicians and nurses in Germany
Source: BMC Palliat Care. 2024 Dec 20;23:292. doi: 10.1186/s12904-024-01625-x (PMC11662584; doi:10.1186/s12904-024-01625-x)
Supplement: Supplementary file 1 — Supplementary Material 1. [file 12904_2024_1625_MOESM1_ESM.docx]

|  | **Number of participants** | **Age** (y) | **m/f** | **Date**  (mm/dd/yyyy) | **Duration**  (min. rounded) | **Presence /online** |
| --- | --- | --- | --- | --- | --- | --- |
| Focusgroups seniors | | | | | | |
| FG 1 | 5 | 75-81 (**⌀** 78) | 3f, 2m | 08/11/2022 | 114 | Presence |
| FG 2 | 5 | 75-84 (**⌀** 79) | 3f, 2m | 08/17/2022 | 113 | Presence |
| FG 3 | 5 | 75-87 (**⌀** 81) | 3f, 2m | 09/21/2022 | 97 | Presence |
| FG 4 | 4 | 75-83 (**⌀** 81) | 3f, 1m | 09/26/2022 | 90 | Presence |
| FG 5 | 4 | 75-83 (**⌀** 79) | 2f, 2m | 10/17/2022 | 85 | Presence |
| FG 6 | 4 | 75-89 (**⌀** 82) | 3f, 1m | 12/02/2022 | 85 | Presence |
| Total: | 27 | 75-89 (**⌀** 83) | 17f, 10m |  | 584 |  |
| Focusgroups physicians | | | | | | |
| FG 1 | 5 | 31-63 (**⌀** 49) | 1f, 4m | 02/22/2023 | 94 | Presence |
| FG 2 | 4 | 28-63 (**⌀** 46) | 4f, 0m | 03/15/2023 | 63 | Presence |
| FG 3 | 3 | 38-51 (**⌀** 46) | 1f, 2m | 03/30/2023 | 75 | online |
| FG 4 | 5 | 40-51 (**⌀** 48) | 4f, 1m | 04/18/2023 | 91 | online |
| FG 5 | 7 | 27-67 (**⌀** 51) | 4f, 3m | 05/25/2023 | 77 | online |
| Total: | 24 | 27-67 (**⌀** 47) | 14f, 10m |  | 400 |  |
| Focusgroups nursing staff | | | | | | |
| FG 1 | 5 | 25-66 (**⌀** 50) | 4f, 1m | 06/30/2022 | 84 | Presence |
| FG 2 | 9 | 26-69 (**⌀** 52) | 5f, 4m | 07/19/2022 | 106 | Presence |
| FG 3 | 5 | 26-55 (**⌀** 37) | 3f, 2m | 07/27/2022 | 95 | online |
| FG 4 | 4 | 25-40 (**⌀** 32) | 3f, 1m | 10/12/2022 | 95 | Presence |
| FG 5 | 5 | 31-58 (**⌀** 51) | 3f, 2m | 01/26/2023 | 105 | Presence |
| Total: | 28 | 25-69 (**⌀** 46) | 18f, 10m |  | 485 |  |
