# Supplementary material for: A ‘good death’ needs good cooperation with health care professionals – a qualitative focus group study with seniors, physicians and nurses in Germany
Source: BMC Palliat Care. 2024 Dec 20;23:292. doi: 10.1186/s12904-024-01625-x (PMC11662584; doi:10.1186/s12904-024-01625-x)
Supplement: Supplementary file 2 — Supplementary Material 2. [file 12904_2024_1625_MOESM2_ESM.docx]

# Focus Group Guide “Medicine in older age” – Seniors

| 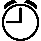 |  | Narrative prompt | Checklist // Memos | Specific questions | Directive and maintaining questions |
| --- | --- | --- | --- | --- | --- |
|  |  | **Welcome** | |  |  |
| 15 minutes |  | Welcome | - - - - Corona-Rules - Audio-recording & pseudonymization - Note: confidentiality - Group rules - Any questions? - Do we need a break? - Introduction round: name, age, motivation for participation | |  |
|  |  | **Information on project** | Project is named “Medicine and the good life in old age”   - We are interested in your attitudes and experiences with medical care - To get closer to this topic, we have prepared two scenarios, we’d like to discuss with you. | | |
| 20 minutes |  | **Case vignette 1: artificial knee joint** | | | |
|  |  | What do you think about this?  What advice would you give Mrs. Malsburg?  How would you decide for yourself? Why? | - Risks of surgery - Social consequences: daughters’ point of view - Guilt / responsibility for her state of health - Right to take risks - Financial aspects: it is “worth it”? |  | Can you explain in more detail why you would make this decision?  Can you put yourself in Mrs. Malsburg’s daughters’ shoes? What would your position be then? |

| 20 minutes |  | Case vignette 2: Feeding via gastric tube | | | | |
| --- | --- | --- | --- | --- | --- | --- |
|  |  | What do you think when you read/hear this?  What advice would you give Mr. Becker?  How would you decide for yourself? Why? | - Quality of life vs. time left - Scarcity of resources (time) - Consequences for family members/loved ones - Risks of PEG tube |  | Can you explain in more detail why you would make this decision?  Can you put yourself in the shoes of Mr. Becker’s relatives? What would a feeding tube mean for them?  Would you advise your father to do the same? | |
| 50 minutes |  | **Senior**’**s own experiences** |  |  |  | |
|  |  | I have the impression that we have collected and discussed the essential aspects of this case.  And now we would like to know: What experiences have you yourself had with medical treatments that have had a positive or negative impact on your everyday life? |  | Which needs are most important in your everyday life?  What problems do you have with them?  What activities would you like to maintain?  What do you want/hope for from your doctor? | What is the first thing that comes to mind when you think of ...?  If discussion is too focused on pos./neg. aspects → specifically ask for neg./pos. examples.  What possibilities/problems emerge from medicine for your personal life? | |
| 10 minutes |  | **Closing** |  |  | |  |
|  |  | It seems that we are slowly coming to an end... / considering the time, we should slowly come to an end.  So, to summarize, we could say that... | - Summary - Did I forget anything? - Any questions? | Questions noted during discussion  Did I forget anything that seems important to you?  Do you want to share anything we didn’t discuss or have not discussed in enough detail? | |  |

Hand out form for expense allowance.

# Focus Group Guide “Medicine in older age” – Physicians & Nursing staff

| 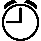 |  | Narrative prompt | Checklist // Memos | Specific questions | Directive and maintaining questions |
| --- | --- | --- | --- | --- | --- |
|  |  | **Welcome** | |  |  |
| 15 minutes |  | Welcome | - - - - Corona-Rules - Audio-recording & pseudonymization - Note: confidentiality - Group rules - Any questions? - Do we need a break?   - - - Introduction round: name, motivation for participation, medical discipline | |  |
|  |  | **Information on project** | Project is named “Medicine and the good life in old age”   - We are interested in your attitudes and experiences with medical care of older adults. - To get closer to this topic, we have prepared two scenarios, we’d like to discuss with you. | | |
| 20 minutes |  | **Case vignette 1: artificial knee joint** | | | |
|  |  | What do you think about this?  What advice would you give Mrs. Malsburg? | - Risks of surgery - Social consequences: daughters' point of view - Guilt / responsibility for her state of health - Right to take risks - Financial aspects: it is “worth it”? |  | Can you explain in more detail why you would decide like this?  What would your decision depend on?  How would you decide if … was different?  Can you put yourself in the shoes of Mrs. Malsburg’s daughters? What would then be your position? |

| 20 minutes |  | Case vignette 2: feeding via gastric tube | | | | |
| --- | --- | --- | --- | --- | --- | --- |
|  |  | What do you think, when you read/hear this?  What advice would you give Mr. Becker? | - Quality of life vs. time left - Scarcity of resources (time) - Consequences for family members/loved ones - Risks of PEG tube |  | Can you explain in more detail why you would decide like this?  What would your decision depend on?  How would you decide, if … was different?  Would you advise your father the same? | |
| 50 minutes |  | **Physicians’ / Nurses’ own experiences** |  |  |  | |
|  |  | I have the impression that we have collected and discussed the essential aspects of this case.  And now we would like to know: What experiences have you yourself had in your everyday professional life? | In your opinion/experience, what needs do seniors have?  What skills should be maintained to continue activities?  What function should medicine have? |  | What is the first thing that comes to your mind when you think of medical care in old age?  If discussion is too focused on pos./neg. aspects → specifically ask for neg./pos. examples.  What opportunities / problems do you think arise from medicine for older people? | |
| 10 minutes |  | **Closing** |  |  | |  |
|  |  | It seems that we are slowly coming to an end... / considering the time, we should slowly come to an end.  So, to summarize, we could say that... | - Summary - Did I forget anything? - Any questions | Questions noted during discussion  Did I forget anything that seems important to you?  Do you want to share anything we didn’t discuss or have not discussed in enough detail? | |  |

Hand out form for expense allowance and certificate of attendance.
