# Supplementary material for: A ‘good death’ needs good cooperation with health care professionals – a qualitative focus group study with seniors, physicians and nurses in Germany
Source: BMC Palliat Care. 2024 Dec 20;23:292. doi: 10.1186/s12904-024-01625-x (PMC11662584; doi:10.1186/s12904-024-01625-x)
Supplement: Supplementary file 3 — Supplementary Material 3. [file 12904_2024_1625_MOESM3_ESM.docx]

**Case vignette 2: feeding via gastric tube**

Gustav Becker (81 years old) is completely dependent on help (eating, washing, dressing) due to his advanced Parkinson's disease (stage 5 according to Hoehn & Yahr)* and early-stage dementia (amnesia)* and lives in a nursing home. He can no longer stand up, it takes a very long time to hand him his food and he often chokes. As a result, he has already been hospitalized three times for pneumonia. The doctors at the hospital advised him to have a PEG tube to feed him. However, Gustav has always loved eating and now sees eating as one of the few pleasures he has left.

What do you think about this?

*Specifications in brackets were for physicians only.
