# Supplementary material for: A ‘good death’ needs good cooperation with health care professionals – a qualitative focus group study with seniors, physicians and nurses in Germany
Source: BMC Palliat Care. 2024 Dec 20;23:292. doi: 10.1186/s12904-024-01625-x (PMC11662584; doi:10.1186/s12904-024-01625-x)
Supplement: Supplementary file 4 — Supplementary Material 4. [file 12904_2024_1625_MOESM4_ESM.docx]

| **Seniors—code tree “dying & death”** | | | |
| --- | --- | --- | --- |
| **No.** | **Main Code** | **Subcode 1^st^ order** | **Memo & coding instructions** |
| **1** | **Preferences for death** |  | Applied only, if no subcode is suitable. |
| 1.1 |  | QOL vs. LST | - Negotiations of QOL vs. remaining lifetime; - Statements, preferences, wishes regarding LST (often reasoned by lack of QOL, for example: approving death to sustain QOL); - Normative claims to admit death of a person; - Negotiations of the right time to die (“being ready to die”); - Negotiations of futile care. |
| 1.2 |  | Places of death | - When places of death addressed; - When good or bad death is illustrated via place of death; - Wanting to die before something happens in order to avoid dying in a certain place (e.g. wanting to die before moving into a nursing home). |
| 1.3 |  | Self-determined death (assisted suicide) | - When self-determined death is addressed; - When assisted suicide is addressed (whether as a wish, a negative or neutral attitude). |
| **2** | **Tensions between acceptance & avoidance** |  | Applied only, if no subcode is suitable. |
| 2.1 |  | Cooperation (team-based decision-making) | - When cooperation with physicians / nurses is addressed; - When medical team is addressed as a factor for (un)successful death; - Communication between professions & organizations. |
| 2.2 |  | Reluctance to deal with own death | - Statements about people not wanting to deal with their own death or with the death of family members and loved ones - Statements about general reluctance in society to deal with the topic of death and its consequences (e.g. lack of knowledge about procedures and possible support) |
| 2.3 |  | (Missing) Own acceptance / inner closure | - Code is applied for positive and negative values (if there is acceptance and if acceptance is lacking). - Acceptance: - Being able to accept one’s own death and let it happen; - Having trouble with accepting one’s own death; - Normative demands that a third person should accept their own death; - Everything related to the process of accepting death (e.g. giving someone time to process dying) or if something hinders this process (e.g. unresolved conflicts). - Inner closure: - “Being ready to die”, having come to terms with life; - Normative claims to “clean up” one’s mental state and social life before death; - Often described in narratives of people who could not die, because there was something left to do that kept them from life completion. |
| 2.4 |  | (Missing) Support by family members | - When family members accept death or wish to die, offer support and accompany a patient’s journey to death; - Missing support by family members (e.g.family members holding back advance directives, because they cannot let go); - When fear is stated that own relatives might not be able to accept one’s own wish to die in the future. |
| 2.5 |  | (Not) Being allowed to die in medicine | - Narratives in which people (and their families) had to fight to be able to die in peace; - When death was prevented by medicine, despite the patient’s (presumed) wish to die; - Positive experiences with medical or nursing staff, e.g. physicians who were supportive in the process of dying (often as an answer to negative experiences); - Assumptions about reasons why a peaceful death can be complicated in the realm of medicine. |
| 2.6 |  | Changes in handling of death | - When, over time, changes in the utilization of palliative care are recognized; - When changes are recognized in the general tendencies to avoid death. |
| **3** | **Consequences for third persons / family members** | | Applied only, if no subcode is suitable. |
| 3.1 |  | Burden/relief of family members | - When the circumstances of death have negative or positive impact on third persons, esp. family members; - When perceptions of family members are in the focus of the narrations and used as a basis to assess the quality of death (e.g. when a death is portrayed as bad, because the deceased’s wife suffered a lot). |
| 3.2 |  | Feelings of guilt | When after the death of a relative feelings of guilt arise in family members, because they feel responsible, e.g. for ending LST. |
| **4** | **Spirituality / Religiosity** |  | - When spiritual or religious questions arise in the context of death (e.g. afterlife); - When the influence of religious/spiritual belief on handling death is addressed; - Code adopts a wide understanding of religious/spiritual belief that covers all sorts of convictions in the context of death (e.g. the belief that one can decide to die at a specific time by the power of will-control). |
| **5** | **Funeral & cemetery** |  | Everything related to funerals, sepulchral culture, care of graves or cemetery (e.g. explanations that arrangements for future tree burial have already been made) |

| **Nursing staff—code tree “dying & death”** | | | | |
| --- | --- | --- | --- | --- |
| **No.** | **Main Code** | **Subcode 1^st^ order** | **Subcode 2^nd^ order** | **Memo & coding instructions** |
| **1** | **Organization of inevitable death** |  |  | Applied only, if no subcode is suitable. |
| 1.1 |  | QOL vs. LST |  | - Negotiations of QOL vs. remaining lifetime; - Negotiations of quality of death of different ways of dying (e.g. not having a gastric tube means starving to death which is an uncomfortable way of dying); - Normative claims to admit death of a person; - Negotiations of the right time to die (“being ready to die”); - Negotiations of futile care. |
| 1.1.1 |  |  | “Reasonable death” | When death is considered comfortable, either due to medical support (palliative care) OR due to rejection of LST in situations with no or little prospect of improvement. |
| 1.1.2 |  |  | “Dirty death” | Prolonged suffering either due to LST or other medical treatment OR due to lack of adequate medical treatment. |
| 1.2 |  | Good support by nurses |  | - When, based on the personal needs and preferences of patients, emotionally competent care by nursing staff was emphasized as positive; - When detecting the patient’s needs regarding a good death was considered important; - When fulfilling the patient’s last wishes was considered important; - Negotiations of institutional differences between organizations and how they affect professional EOL support (e.g. in a nursing home, nurses know the patients and their preferences better than in a hospital and can therefore provide a more personal kind of care). |
| 1.3 |  | Places of death |  | All statements regarding places of death. For instance, this may be:   - Opinions about how places of death influence quality of death (e.g. in hospital, it’s not possible to die in peace); - Statements about where people want to die; - Experiences or ideas of what conditions prevail in specific places of death and their influence on care options. |
| **2** | **Decision-making authorities at the EOL** |  |  | Applied only, if no subcode is suitable. |
| 2.1 |  | Self-determination / deciding for oneself |  | - Expressions of opinion that EOL decisions can only be made individually but not for third persons; - Statements that patients need to be given the opportunity to decide for themselves; - When it was noted that EOL decisions need to consider the highly intimate and individual nature of death. |
| 2.2 |  | Decision / opinion of family members |  | - Views that family members, morally or legally, must be included in EOL decisions; - When situations were reported in which family members made EOL decisions or the participants themselves had to make EOL decisions as family members, and, if applicable, what difficulties they faced in these situations. |
| 2.3 |  | Decision against patient’s wishes |  | Experiences of situations in which physicians and/or family members made decisions contradicting the patient’s wishes (e.g. a wife holding back her husband’s advance directive, because she cannot bear to see him die); |
| 2.4 |  | Preparation for death |  | Observations that preparation for death is necessary in order to avoid a patient’s hospital admissions despite palliative care in a nursing home being more appropriate. |
| 2.5 |  | Role and relation of professionals |  | - When the role of professionals in EOL care is addressed; - When the relation between nurses and physicians is addressed and the text passage is not clearly be assigned to one of the two subcodes (e.g. when the view is taken that nurses must bear medical decisions and their consequences without being involved in the decision-making process and nurses feel burdened by the physician’s decision-making power). |
| 2.5.1 |  |  | Self-perception of nurses | Statements about role, opinion and self-perception of nurses with regard to dealing with patients at the EOL (e.g. when the view is taken that nurses know patients and their needs better than physicians, because they spend more time with them). |
| 2.5.2 |  |  | Decision-making power of physicians | - When physician’s opportunities to influence patients within informed consent discussions are addressed; - Negotiations of limitations of physician’s decision-making power; - When legal predominance of physician’s decisions is addressed; - When physician’s responsibility to deal with their decision-making power is addressed. |
| **3** | **Avoidance and difficulties** |  |  | Applied only, if no subcode is suitable. |
| 3.1 |  | Death as institutional avoidance tendency |  | - Reports of organizational procedures or treatment cultures in the hospital / nursing home that are by default designed to preserve and prolong life (often as narratives of “in the hospital, death is not allowed”); - When such institutional avoidance tendencies are observed to be performed by physicians or nurses and are attributed to specific reasons (e.g. financial interests, fear of liability, lack of knowledge/training, etc.). |
| 3.2 |  | Death as social avoidance tendency |  | - When it is argued that dealing with one’s own imminent death is uncomfortable and avoided (in the sense of repression); - Opinions that people usually avoid to confront family members and loved ones with the topic of death and that it is challenging to speak about death within families; - Opinions about societal avoidance of death (e.g. general level of knowledge in society is low, because people don’t want to deal with death). |
| 3.2.1 |  |  | Family members cannot “let go” | When situations had been experienced in which family members were not able to let go, possibly with the result that medical measures were applied against the patient’s wishes or death was prevented, although the patient wanted to die. |
| 3.3 |  | Not wanting / being able to decide |  | - When EOL decisions are generally understood as a burden for the one’s having to decide; - When patient’s, family members, or health care professionals are unable to make a decision, because the decision is too heavy a burden (e.g. story about a patient who clearly wanted their children to make EOL dicisions, but the children clearly state that they feel unable to do so and request the physicians to make the decisions). |
| 3.3 |  | Changes in handling of death |  | - When it is described that the way society and/or medicine deals with death has changed and is changing; - Often in form of stories about how society and medical institutions have reduced their level of avoidance and allow death to happen more easily/frequently. |
| 3.4 |  | Feelings of guilt |  | Thematization of legal guilt or personal feelings of guilt that can occur in formal or informal caretakers after the death of a person. |
| **4** | **(Non-)assisted suicide** |  |  | When the topic of suicide is raised and the desire for assistance that goes beyond palliative care is discussed. |

| **Physicians—code tree “dying & death”** | | | | |
| --- | --- | --- | --- | --- |
| **No.** | **Main Code** | **Subcode 1^st^ order** | **Subcode 2^nd^ order** | **Memo & coding instructions** |
| **1** | **QOL and life extension** |  |  |  |
| 1.2 |  | QOL vs. LST |  | - Negotiations of QOL vs. remaining lifetime; - Negotiations of lack of QOL, which could be retrieved by medical means / LST; - Negotiations of determinants of QOL, especially social integration and life goals (e.g. to live to see great-grandchildren being born or grandchildren being sent to school); - Often overlapping with code “2.4 Call for SDM” due to discussion of patient involvement in these negotiations. |
| 1.2.1 |  |  | Palliative medicine | - When palliative care is mentioned; - Considerations of opportunities of palliative care; - When specific characteristics or attitudes of palliative care compared to other disciplines are discussed; - When palliative care is discussed as an alternative to curative treatment or assisted suicide. |
| 1.2.2 |  |  | Is intensive care justified at high age? | When it is discussed whether there is a limit at which it can justifiably be said that treatment in the ICU at this age and with this health status is no longer appropriate and should no longer take place, even if the patients or relatives may still want it to happen. |
| 1.2.3 |  |  | Death as salvation | When the death of a person is seen by third parties as salvation. |
| 1.3 |  | Places of death |  | - When place of death is mentioned or discussed (usually happens casually or concerning transfer to hospital); - Considerations on the extent to which the place of death influences the circumstances of death. |
| 1.4 |  | Voluntary stopping of eating and drinking (VSED) |  | Mentioning / Discussions of voluntary stopping of eating and drinking in order to pursue the desire to die. |
| 1.5 |  | Assisted suicide |  | When assisted suicide is mentioned or discussed. |
| **2** | **Finding consent & treatment decisions** |  |  | Applied only, if no subcode is suitable. |
| 2.1 |  | Role of physicians in medical decision-making |  | Discussions of physician’s role and responsibilities in medical decision-making at the EOL (e.g. responsibility of physicians to ask patients’ family members about patients’ wishes and then make a decision). |
| 2.1.1 |  |  | Self-perception of physicians as decision makers | - Physicians’ self-perception as key players in EOL treatment decisions (including through the influence that physicians automatically exert through informed consent discussions); - Problems and difficulties with such decisions specifically related to the physicians position (e.g. seeing nursing home patients only in large intervals). |
| 2.1.2 |  |  | Differences across disciplines | When different perspectives from different disciplines or the difference between outpatient and inpatient care influence are reflected or said to influence medical treatment decisions at the EOL. |
| 2.1.3 |  |  | Personal experiences / opinions of physicians | When it is discussed that personal attitudes or (private and professional) experiences influence medical decision-making (e.g. experienced death of own parents leads to increased sensitivity for the pitfalls in EOL care). |
| 2.2 |  | Role of family members |  | Applied only, if no subcode is suitable. |
| 2.2.1 |  |  | Family members determine patient’s wishes and decide | - When it is suggested that relatives should be involved as key decision-makers (often: “they know the presumed will best”); - When this approach is critized (e.g. relatives should not be burdened with decisions, because treatment decisions are a medical task; relatives should only help to determine the presumed will). |
| 2.2.2 |  |  | Decision for the sake / in the interests of family members | When patients at the EOL base their decisions about how to proceed with treatment largely on the feelings of their relatives, often in connection with “not wanting to be a burden” (e.g. when a patient who wants to die decide to further utilize LST, because their relatives want them to live). |
| 2.2.3 |  |  | Saying goodbye / emotional support | - The view that relatives want to say goodbye and are those who accompany death; - The fact that relatives can be present can be a value in itself or make for a good death. |
| 2.3 |  | Preparation for death |  | - All mentioned forms of advance planning, advance directives, discussions of EOL issues with family in advance, etc.; - Normative requests that patients deal with upcoming issues and have already thought about them; - Emphasis on physician’s own responsibility to initiate this form of reflection and advance planning; - criticism of colleagues or organizations like nursing homes that did not act in advance and initiate these discussions, e.g. to prevent readmission to hospital; - sometimes overlaps with code “demand for SDM”. |
| 2.4 |  | Call for SDM |  | When it is requested to discuss the patient’s current health situation, possible treatment options and their potential consequences in detail and to understand and acknowledge patient’s motivations and opinions. |
| 2.4.1 |  |  | Presumed Patient wishes | Discussions about how to assess a patient’s wishes when the patient is not able to express them. |
| 2.5 |  | Informed consent discussions are pointless |  | If the benefit of informed consent discussions is doubted because patients are either no longer cognitively able to follow such discussions or the information is perceived as worthless/unhelpful for the individual’s decision. |
| 2.6 |  | Cooperation (team-based decision-making) |  | - When it is stated that decisions can be made better in a team; - Discussions about team members who have to bear the consequences of medical decisions; - “Team” ranges from medical team in inpatient settings to cooperation between different HCP from in- or/and outpatient care. |
| **3** | **Tabuisierung / Verdrängung des Sterbens** |  |  | Applied only, if no subcode is suitable. |
| 3.1 |  | Physicians cannot allow death |  | Narratives of situations in which physicians have found it (particularly) difficult to let someone die (for reasons of medical attitude or professional ethos). |
| 3.2 |  | Technological imperative |  | - When technological achievements (e.g. LST) are considered in comparison to the “state of nature” in order to see or evaluate them from a different perspective; - When it is discussed that it can be challenging to refuse available treatment; - When it is questioned whether technological possibilities are cultural achievements and must therefore be applied; - Discussions about expectations that are placed on medicine that there is still something that can and should be done, when in reality medicine cannot do anything anymore. |
| 3.3 |  | Death as social avoidance tendency |  | - When it is argued that dealing with one’s own imminent death is uncomfortable and avoided (in the sense of repression); - Opinions that people usually avoid to confront family members and loved ones with the topic of death and that it is challenging to speak about death within families; - Opinions about societal avoidance of death (e.g. general level of knowledge in society is low, because people don’t want to deal with death). |
| 3.3.1 |  |  | Family members cannot “let go” | Reports of relatives that find it challenging to let the patient go or that even take steps to prevent/delay death, even though this does not correspond to the (presumed) will of the patient. |
| 3.3.2 |  |  | Patients cannot accept their own death | When patients cannot/will not accept their own imminent death and either deny it or demand more and more curative measures. |
| 3.4 |  | Changes in handling of death |  | - When changes are recognized in the general tendencies to avoid death (both in everday life and in medical sphere) (can be observed, e.g., in the increased desire to die at home); - When an increasing public or professional debate on the utilization of LST is observed. |
| **4** | **Feelings of guilt** |  |  | When the feeling of guilt about someone's death is addressed, regardless of who feels this guilt or for whatever reasons. |
| **5** | **Religious / cultural aspects** |  |  | When it is discussed that religious aspects or cultural background shape the way in which death is handled and the wishes of patients and their family members and should be taken into account. |
